# Ultrasound-Assisted Emulsification Microextraction Based on Solidification Floating Organic Drop Trace Amounts of Manganese Prior to Graphite Furnace Atomic Absorption Spectrometry Determination

**DOI:** 10.1100/2012/987645

**Published:** 2012-05-02

**Authors:** Alireza Mohadesi, Masoumeh Falahnejad

**Affiliations:** Department of Chemistry, Payame Noor University, P.O. Box 19395-3697, Tehran, Iran

## Abstract

In the present study, an ultrasound-assisted emulsification microextraction based on solidification floating organic drop method is described for preconcentration of trace amounts of Mn (II). 2-(5-Bromo-2-pyridylazo)-5 diethylaminophenol was added to a solution of Mn^+2^ at ph = 10.0. After this, 1-undecanol was added to the solution as an extraction solvent, and solution was stirred. Several factors influencing the microextraction efficiency, such as pH, the amount of chelating agent, nature and volume of extraction solvent, the volume of sample solution, stirring rate, and extraction time were investigated and optimized. Then sample vial was cooled by inserting into an ice bath, and the solidified was transferred into a suitable vial for immediate melting. Finally the sample was injected into a graphite furnace atomic absorption spectrometry. Under the optimum condition the linear dynamic range was 0.50–10.0 ng mL^−1^ with a correlation coefficient of 0.9926, and the detection limit of 0.3 ng mL^−1^ was obtained. The enrichment factor was 160. The proposed method was successfully applied for separation and determination of manganese in sea, rain, tap, and river water samples.

## 1. Introduction

Manganese is recognized as both an essential and a neurotoxic trace element. As an essential trace element, Mn plays an important role in bone and tissue formation, reproductive functions, and the activation of many enzymes, which are involved in vital metabolic processes [1]. Element deficiency is not a common occurrence since dietary sources provide an adequate supply of 2–8 mg of Mn per day. However, toxic levels may be reached in workers or individuals living near mines, ore-processing plants, or manufactures of varnish, pharmaceutical products, ceramics, and pottery. There is still little information on the biochemical mechanism, which could explain the like symptoms, caused by chronic inhalation of excess levels of Mn [1, 2]. Manganese exists mainly in both manganese (II) and manganese (IV) oxidation states in ordinary aqueous environments. In aqueous environments manganese (IV) is a dominant chemical species and exists in insoluble forms, such as particulate and colloidal MnO_2_. However, manganese (II) ion is rather stable in aqueous environments, which are often linked with water pollution, especially for drinking water [3, 4]. The greatest parts of dissolved manganese in environmental waters are thought to be manganese (II) ion [5]. The direct determination of trace manganese ions is generally difficult because of matrix interference problems and low concentration of metals in samples. These problems can be overcome by using preconcentration and separation procedures before the detection procedure. For this purpose, various methods for the separation and preconcentration of manganese have been reported, such as solid-phase extraction [6–10], liquid-liquid extraction [11], cloud point extraction [12], and liquid membranes [13] have been widely used. Recently a new liquid-liquid microextraction method based on solidification of floating organic drop which was successfully used for the extraction and determination of lead, cooper, palladium, cobalt, and nickel was reported [14–17]. In this method, small volume of an organic solvent with a melting point near room temperature (in the range of 10–30°C) was floated on the surface of aqueous solution. The aqueous phase was stirred for a prescribed period of time, and then the sample was transferred into the ice bath. When the organic solvent was solidified, it was transferred into a small conical vial, and the melted organic solvent was used for analytes determination. The proposed method is very simple and inexpensive. In this method the enrichment factor is higher than the reported methods such as solid phase extraction, liquid-liquid extraction, and cloud point extraction. The LOD of this method is lower, and the extraction time of this method is shorter than that of the other methods mentioned above. In addition, this method uses less toxic organic solvent and it is one of the most important advantages of this method [18]. In this study we consider the possibility of implementation of ultrasound-assisted emulsification microextraction based on solidification floating organic drop (USA-EME-SFO) in combination with graphite furnace atomic absorption spectrometry (GFAAS) in trace manganese analysis. The applicability of the approach was demonstrated for the determination of manganese in water samples. The influence of different experimental parameters on the recovery of the extraction, such as solution pH, chelating agent amount, volume of extraction solvent, and salt effect was described and discussed. Operation simplicity, rapidity, short extraction time, using less amount of toxic organic solvent, and high enrichment factor are some of the USA-EME-SFO advantages.

## 2. Experimental

### 2.1. Instrumentation

A SpectrAA 220 atomic absorption spectrometer equipped with a graphite furnace atomizer (GTA-110 series) and manganese hollow cathode lamp were used for absorbance measurements at wavelength of 279.5 nm according to instrument instruction. The instrumental parameters were adjusted according to the manufacturer's recommendations. The sample injection volume was 10.0 *μ*L in all experiments. The pH values were measured with a Metrohm pH meter (Model: 692 Metrohm, Switzerland), supplied with a glass-combined electrode. A NAPCO 2028R Centrifuge was used for centrifugation. An ultrasonic bath system (Model: Tecna 6, Italy) was used for cloudy mixture formation. All 10.0 mL screw cap falcon test tubes were maintained into 0.1 mol L^−1^ HNO_3_ for cleaning any inorganic compounds and washed with deionized water and then with acetone for proper sedimentation of fine droplets of the extraction solvent in the centrifugation step.

### 2.2. Reagents and Solutions

Ultrapure water was used throughout the work. 2-(5-Bromo-2-pyridylazo)-5 diethylaminophenol (5-Br-PADAP) and 1-undecanol were from Merck company (Darmstadt, Germany). Water samples were filtered through 0.45 *μ*m membrane filters (Millipore), and then pH of these samples was adjusted to 10.0 with NH_4_Cl/NH_3_ buffer (pH*∼*10). The stock standard solution for Mn was prepared immediately before use, by stepwise dilution of 1000.0 *μ*g mL^−1^ Mn (II) stock standard solution in HNO_3_ 0.5 mol L^−1^.

### 2.3. Sample Preparation

Sea, rain, river, and tap water samples were collected in acid-leached polyethylene vials. Acidification to pH 1.0 with nitric acid was performed immediately after collection, in order to prevent adsorption of the manganese ions on the vial walls. The samples were filtered before analyses through a cellulose membrane (Millipore) of 0.45 *μ*m pore size. 

### 2.4. Ultrasound-Assisted Emulsification Microextraction Based on Solidification Floating Organic Drop Procedure

Aliquots of sample solution were placed into a 10 mL vial, and then 200.0 *μ*L of 5-Br-PADAP 0.05% and 0.8 mL NaCl 10% were added to it as a chelating agent and salt, respectively. The solution diluted to 8 mL, and solution pH adjusted at 10.0 with NH_4_Cl/NH_3_ buffer. Then 50.0 *μ*L of 1-undecanol was injected to the solution using a 1.0 mL microsyringe. The sample solution was sonicated for 5 min until the cloudy mixture was formed. Then the mixture was centrifuged at 6000 rpm for 10 min. Finally vial was transferred into an ice bath. The solidified organic solvent was transferred into the conical vial. After melting the solvent, 10.0 *μ*L of this melted solvent was injected into the GFAAS for quantification. 

## 3. Results and Discussion

### 3.1. Optimization of Furnace Temperature Program

 Preliminary studies of the behavior in GFAAS of an extract of standard solution with the temperature program recommended by manufacturers demonstrated that the magnitude of background signal was high. In order to reduce the background without losing the manganese, the temperature program of the furnace was optimized, and the final results are given in Table 1. Under these conditions the background is low and manganese peak has a normal shape. 

### 3.2. Effect of pH

 The pH plays a unique role on metal-chelate formation and subsequent extraction. So the effect of pH on the USA-EME-SFO of Mn (II) was studied in the pH range of 1.0–10.9 using nitric acid and sodium hydroxide with keeping the other variables constant. The results demonstrated that the recovery is nearly constant in the pH range of 9.2–10.5, so pH 10.0 was selected for further experiments. 

### 3.3. Effect of Chelating Agent Amount

 The influence of the amount of 5-Br-PADAP was also evaluated, and the results showed that the maximum recovery is obtained with 200 *μ*L of 0.05% 5-Br-PADAP solution. 

### 3.4. Nature and Volume of Extraction Solvent Effect

The organic solvent used as the extraction solvent in this method should satisfy several criteria: (1) it should have lower density than water; (2) it should be low volatile to prevent loss of the solvent during the extraction process; (3) it should provide an appropriate extraction efficiency to provide high extraction recoveries and thus high enrichment factor; (4) its melting point should be near the room temperature (10–30°C) [24]. Accordingly, several extraction solvents such as 1-undecanol, 1-dodecanol, and 2-dodecanol were investigated. Based on the obtained results, 1-undecanol had the best extraction efficiency. Also because of its low vapor pressure at the extraction conditions, the extract was stable at the extraction period. Therefore, 1-undecanol was selected as the extraction solvent. The effect of extraction solvent volumes on the recovery was determined, in the range of 30.0–90.0 *μ*L. Results showed that 50.0 *μ*L is the optimum volume of 1-undecanol. 

### 3.5. Effect of Extraction Time

The influence of the extraction time was examined in the experimental conditions. In USA-EME-SFO, extraction time is defined as the time interval between injecting of the extraction solvent and starting to centrifuge. The results displayed that the extraction time had no notable effect on the recovery of extraction, so 5 min was selected as an extraction time in this procedure.

### 3.6. Salt Effect

For investigating the influence of the ionic strength on the USA-EME-SFO performance, several experiments were performed by adding different kinds of salt, such as KI, NaCl, and NaNO_3_. The recoveries were 75.8%, 98.6%, and 62.1% for KI, NaCl, and NaNO_3_, respectively. NaCl was selected for subsequent experiments. The effect of the amount of NaCl on the extraction efficiency was studied with different amount of NaCl in the range of 0.01–0.3 g NaCl. 

### 3.7. Effect of Diverse Ions

Various salts and metal ions were added to a solution containing 3.0 ng mL^−1^ of Mn (II) ions, and the general procedure was applied. The results (error < ±5%) are given in Table 2. most of the metal ions that were studied did not interfere higher than 500 mol ratio level. Thus the method is selective and may safely be applied for the determination of manganese in various water samples.

### 3.8. Figures of Merit

 Performance characteristics of the method were obtained by processing standard solution of manganese. For a sample volume of 8 mL, the calibration graph exhibited linearity over the range of 0.5–10 ng mL^−1^ with a correlation coefficient of 0.9926. The relative standard deviation (*n* = 8) at 3 ng mL^−1^ was ±3.3%. The enrichment factor for the proposed method was 160, as obtained from the ratio of the volume of the aqueous phase to organic phase. The limit of detection, based on two times the standard deviation of the blank signal divided by the slop of the calibration curve, was 0.3 ng mL^−1^. 

### 3.9. Water Analysis

The proposed method was applied to the determination of Mn (II) in different water samples, and the results along with the recovery for the spiked samples were given in Table 3. As could be seen, the recoveries for the three spiked water samples were in the range of 97.5–105%. 

### 3.10. Comparison of USA-EME-SFO with Other Methods

 A comparison of the represented method with other reported preconcentration methods of Mn (II) is given in Table 4. As can be seen, the present method has higher enrichment factor than other methods such as cloud point extraction (CPE) [19], single-drop microextraction (SDME) [20], and dispersive liquid-liquid microextraction (DLLME) [21]. This method also has a lower limit of detection than CPE [19], solid phase extraction (SPE) [22], ligandless-ultrasound-assisted emulsification microextraction (LL-USAEME) [23], and DLLME [21] methods. Thus it is suitable for ultra trace analysis of manganese in aqueous samples. 

## 4. Conclusions

This paper outlines a successful development and application of the USA-EME-SFO technique, combined with the GFAAS, for trace determination of manganese in several categories of water samples. Compared with other conventional sample preparation methods, the analytical technique offered numerous advantages such as simplicity, low cost, ease of operation, rapid analysis time, and reproducible and high enrichment factor and is suitable for determining manganese in different water samples. The extraction solvent (1-Undecanol) of this method has lower toxicity than DLLME, and thus this method is more environmental friendly.

## Figures and Tables

**Table 1:** Temperature program of GFAAS for determination of manganese.

Steps	Temperature (°C)	Time (S)	Argon flow rate (L min^−1^)	Read command
Dry stage	85	5.0	3.0	No
Dry stage	95	40.0	3.0	No
Dry stage	270	10.0	3.0	No
Ash stage	700	5.0	3.0	No
Ash stage	700	1.0	3.0	No
Gas stop step	700	2.0	0.0	No
Ramp step and read command	2400	1.1	0.0	Yes
Atomize hold step and read command	2400	2.0	0.0	Yes
Tube clean with maximum gas flow	2430	2.0	3.0	No

**Table 2:** Effect of diverse ions.

Interferent	Interferent/Mn (II) ratio (mol/mol)	Recovery (%)
Fe^3+^	10000	96.3
Ca^2+^, Mg^2+^	5000	95.9
Zn^2+^	4500	97.6
Cd^2+^	2000	97.1
Al^3+^	2000	96.7
Ni^2+^	1000	95.6
Pb^2+^, Cu^2+^	800	95.8
Co^2+^	500	95.4

**Table 3:** Determination of manganese in the water samples.

Sample	Spiked (ng mL^−1^)	Found^a^ (ng mL^−1^)	Recovery (%)
Sea water (Persian gulf)	0.00 0.20	7.60 ± 0.32 7.79 ± 0.28	— 95.0
Rain water (Kerman)	0.00 0.70	No detect 0.74 ± 0.03	— 105.7
Tap water (Tehran)	0.00 0.20	5.24 ± 0.21 5.45 ± 0.24	— 105.0
Tap water (Kerman)	0.00 0.30	1.94 ± 0.07 2.23 ± 0.08	— 96.6
River water (Esfahan)	0.00 0.60	3.28 3.90	— 103.3

^a^Mean ± Standard deviation (*n* = 3).

**Table 4:** Comparison of the USA-EME-SFO with other methods for extraction and determination of manganese.

Method	Enrichment factor	LOD (ng·mL^−1^)	Reference
LLE using water-in-oil emulsion-AAS	820	0.02	[12]
CPE-FAAS	20	1.4	[19]
SDME-ETAAS	30.3	0.02	[20]
DLLME-FAAS	82.6	0.5	[21]
SPE-FAAS	—	5	[22]
LL-USAEME-FAAS	—	0.5	[23]
USA-DLLME-SFO-GFAAS	160	0.30	This work
